# Supplementary material for: Durability of the insecticidal activity of next-generation insecticide treated nets distributed for malaria control in Mozambique: Findings from the New Nets Project (2020–2022)
Source: PLOS Glob Public Health. 2026 Jun 5;6(6):e0005306. doi: 10.1371/journal.pgph.0005306 (PMC13240886; doi:10.1371/journal.pgph.0005306)
Supplement: S1 Table — The table describes the mosquito strains used, primary test methods, key outcome measures, and any additional tests conducted for each product type. (DOCX) [file pgph.0005306.s001.docx]

**Durability of the insecticidal activity of next-generation insecticide treated nets distributed for malaria control in Mozambique: findings from the New Nets Project (2020–2022)**

**Authors:** Josias Fagbohoun^1,2,3^, Ana Paula Abílio^4^, Boris N’Dombidje^1,2,3,^, Damien Todjinou^1,2,3^, Marie Baes^5^, Olivier Pigeon^5^, Germain Gil Padonou^1^, Christen Fornadel^6^, Baltazar Candrinho^4,7^, Hannah Koenker^8^, Molly Robertson^9‡^, Joseph Wagman^9^, Corine Ngufor^1, 2, 3,10*^,

**Table S1.** Summary of bioassay methods, mosquito strains, and outcome measures used to evaluate ITN bioefficacy by net type and active ingredient

| **ITN type** | **Brand** | **Active ingredient** | **Strain** | **Primary Test Method** | **Key outcome measures** | **Remarks/additional tests** |
| --- | --- | --- | --- | --- | --- | --- |
| Pyrethroid-only | MAGNet®, Olyset®, DuraNet® | Alpha-cypermethrin | KISUMU-S | Cone bioassays | Knockdown, 24h Mortality | Tunnels for failed nets |
| Pyrethroid-only | Olyset® Net | Permethrin | KISUMU-S | Cone bioassays | Knockdown, 24h Mortality | Tunnels for failed nets |
| Pyrethroid-PBO | Olyset® Plus | Permethrin | KISUMU-S | Cone bioassays | Knockdown, 24h Mortality | Tunnels for failed nets |
|  |  | PBO | AKRON-R | Tunnel tests | 24h Mortality | - |
| Pyrethroid-chlorfenapyr | Interceptor® G2* | chlorfenapyr | VKPER-R | Tunnel tests | 72h Mortality, BFI (alpha) | - |
| Pyrethroid-pyriproxyfen | Royal Guard® | Alpha-cypermethrin | KISUMU-S | Cone bioassays | Knockdown, Mortality | Tunnels for failed nets |
|  |  | Pyriproxyfen | Blood-fed AKRON-R | Cone bioassays | Reduction in Fertility (dissection) | >30% fertility in control Assumed 50% cut off |

**Interceptor® G2 nets were tested in hut trials prior to bioassays*
