# Supplementary material for: Durability of the insecticidal activity of next-generation insecticide treated nets distributed for malaria control in Mozambique: Findings from the New Nets Project (2020–2022)
Source: PLOS Glob Public Health. 2026 Jun 5;6(6):e0005306. doi: 10.1371/journal.pgph.0005306 (PMC13240886; doi:10.1371/journal.pgph.0005306)
Supplement: S2 Table — Outcomes include mosquito deterrence, exophily, blood-feeding, blood-feeding inhibition, 72-hour mortality, and corrected mortality against wild pyrethroid-resistant Anopheles gambiae s.l. populations. (DOCX) [file pgph.0005306.s002.docx]

**Supplementary information**

**Durability of the insecticidal activity of next-generation insecticide treated nets distributed for malaria control in Mozambique: findings from the New Nets Project (2020–2022)**

**Table S1: Detailed results from experimental hut trial with new and field aged Interceptor® G2 nets**

|  |  | **Untreated net** | | **MagNet New** | | **Interceptor New** | | **Interceptor G2 New net** | | **Interceptor G2 Mozambique** | |
| --- | --- | --- | --- | --- | --- | --- | --- | --- | --- | --- | --- |
|  |  | 6 months | 24 months | 6 months | 24 months | 6 months | 24 months | 6 months | 24 months | 6 months | 24 months |
| Deterrency | Total females caught | 241 | 268 | 257 | 648 | 118 | 200 | 166 | 73 | 141 | 214 |
|  | % Deterrence | - | - | -6.6 | -141.8 | 51.0 | 25.4 | 38.1 | 69.7 | 41.5 | 20.1 |
| Exophily | Total veranda | 116 | 98 | 138 | 365 | 61 | 106 | 111 | 40 | 103 | 124 |
|  | % Exophily | 48.1 | 36.6 | 53.7 | 56.3 | 51.7 | 53.0 | 66.9 | 54.8 | 73.0 | 57.9 |
|  | 95% Conf. limits | 41.82-54.44 | 30.80-42.33 | 47.60-59.79 | 52.51-60.15 | 42.68-60.71 | 46.08-59.92 | 59.71-74.03 | 43.38-66.21 | 65.73-80.37 | 51.33-64.56 |
| Blood feeding | Total blood-fed | 110 | 165 | 87 | 297 | 43 | 97 | 64 | 21 | 18 | 107 |
|  | % Blood-fed | 45.6 | 61.6 | 33.9 | 45.8 | 36.4 | 48.5 | 38.6 | 28.8 | 12.8 | 50.0 |
|  | 95% Conf. limits | 39.35-51.93 | 55.74-67.39 | 28.07-39.64 | 42.00-49.67 | 27.76-45.12 | 41.57-55.43 | 31.15-45.96 | 18.38-39.15 | 7.26-18.27 | 43.30-56.70 |
|  | % Blood-feeding inhibition | - | - | 25.8 | 25.6 | 20.2 | 21.2 | 37.4 | 37.0 | 72.0 | 18.8 |
| Mortality | Total 72 h mortality | 4 | 2 | 37 | 125 | 21 | 38 | 112 | 43 | 78 | 83 |
|  | % 72 h mortality | 1.7 | 0.7 | 14.4 | 19.3 | 17.8 | 19.0 | 67.5 | 58.9 | 55.3 | 38.8 |
|  | 95% Conf. limits | 0.05-3.27 | -0.28-1.78 | 10.10-18.69 | 16.25-22.33 | 10.90-24.70 | 13.56-24.44 | 60.34-74.60 | 47.62-70.19 | 47.11-63.53 | 32.26-45.31 |
|  | % corrected 72 h mortality | - | - | 13.0 | 2.0 | 16.4 | 4.5 | 6.2 | 58.2 | 54.6 | 5.6 |
|  |  |  |  |  |  |  |  |  |  |  |  |
